# Complete mitochondrial genome of xanthid crab *Atergatis floridus* (Linnaeus, 1767)

**DOI:** 10.1080/23802359.2018.1437832

**Published:** 2018-02-10

**Authors:** Mustafa Zafer Karagozlu, Michelle M. Barbon, Thinh Do Dinh, Cesar G. Demayo, Chang-Bae Kim

**Affiliations:** a Department of Biotechnology, Sangmyung University, Seoul, Korea;; b Department of Biological Sciences, College of Science and Mathematics, Mindanao State University, Iligan Institute of Technology, Iligan City, Philippines

**Keywords:** Arthropoda, Decapoda, Xanthidae, complete mitochondrial genome, *Atergatis floridus*

## Abstract

The complete mitochondrial genome sequenced from the floral egg crab *Atergatis floridus* (Linnaeus, 1767) and the determination of the position of the species in the reconstructed phylogenetic tree of the infraorder Brachyura using the protein coding mitochondrial genes are presented. Results show the mitochondrial genome length of *A. floridus* is 16,435 bp with nucleotide distribution as 33.4% A, 20.3% C, 10.5% G and 35.8% T. The structure of the complete mitochondrial genome of the species is the same as with the previous xanthid record. The result of the phylogenetic analysis suggests that *A. floridus* is the closest species to other Xanthidae species in the brachyuran records. This is the first complete mitochondrial genome record from the genus *Atergatis*.

The floral egg crab *Atergatis floridus* (Linnaeus 1767) is a cosmopolitan xanthid crab that is highly distributed in the Indo-Pacific region (Ng and Davie 2007). It is known to accumulate high levels of neurotoxins in its cheliped muscles (Arakawa et al. 1995; Saito et al. 2006), even in its exoskeleton and viscera (Inoue et al. 1968; Asakawa et al. 2014). In this study, the complete mitochondrial genome of *A. floridus* was analyzed and molecular phylogeny of the Xanthidae family in the infraorder Brachyura was investigated based on protein coding genes of the mitochondrial genome.

The *A. floridus* species was collected in the shores of Brgy. Amontay, Nasipit, Agusan del Norte/Philippines (8°59′25.98″N 25°19′17.97″E) on April 2017 and immediately preserved in 97% ethanol. After extraction of whole genomic DNA from the cheliped muscle, complete mitochondrial DNA analysis and the reconstruction of phylogenetic tree methods processed by following the study described previously (Karagozlu et al. 2016). The specimen was deposited in the Department of Biotechnology, Sangmyung University, Korea (SM00249).

The size of the complete mitochondrial genome of *A. floridus* was 16,180 bp (GenBank accession number: MG792341). It consists of 13 protein coding genes (PCGs), two ribosomal RNA genes, and 22 tRNAs genes. Among them 23 genes (nine PCGs and fourteen tRNAs) encoded on the majority strand and the remaining 14 genes (four PCG, eight tRNAs and two rRNAs) were encoded in the minority strand which is typical for a brachyuran species. There were three initiation codons used for protein synthesis in the mitochondrial genome namely, ATG, ATT and ATA. ATG was the most common in the protein coding genes. It is used by eight different genes (*atp8*, *cox1*, *cox2*, *cox3*, *cytb*, *nad2*, *nad4* and *nad4l*). On the other hand, ATA was only used by the *atp6* gene. As stopping codons TAA, TAG and incomplete T(AA) were used in the mitochondrial genome. The most common stopping codon identified was TAA. TAG was used by two genes (*atp8* and *nad2*) while T(AA) was only used by the *cytb* gene.

To determine the position of *A. floridus* in the phylogeny of brachyuran crabs, the phylogenetic tree of the different identified species were reconstructed based on the complete mitochondrial records of brachyuran species (maximum three species per superfamily) retrieved from the GenBank (Figure 1). The mitochondrial genome record of *Leptodius sanguineus* (KT896744) was the only complete record from the xanthid crab species and was determined to be the closest to *A. floridus.* The results also showed the closest superfamily to Xanthoidae was Bythograeoidea. It is important to note that based from the GenBank records, the species *Pseudocarcinus gigas* (AY562127) belongs to the superfamily Xanthoidae. However, It was found that the species belong to the superfamily Eriphoidae thus it is suggested that there is a need for the revision in the label of this record as (WoRMS 2018). The present study is the first to show a complete mitochondrial genome from the genus *Atergatis* thus contributes to the strengthening of the brachyuran molecular genome library.

**Figure 1.**
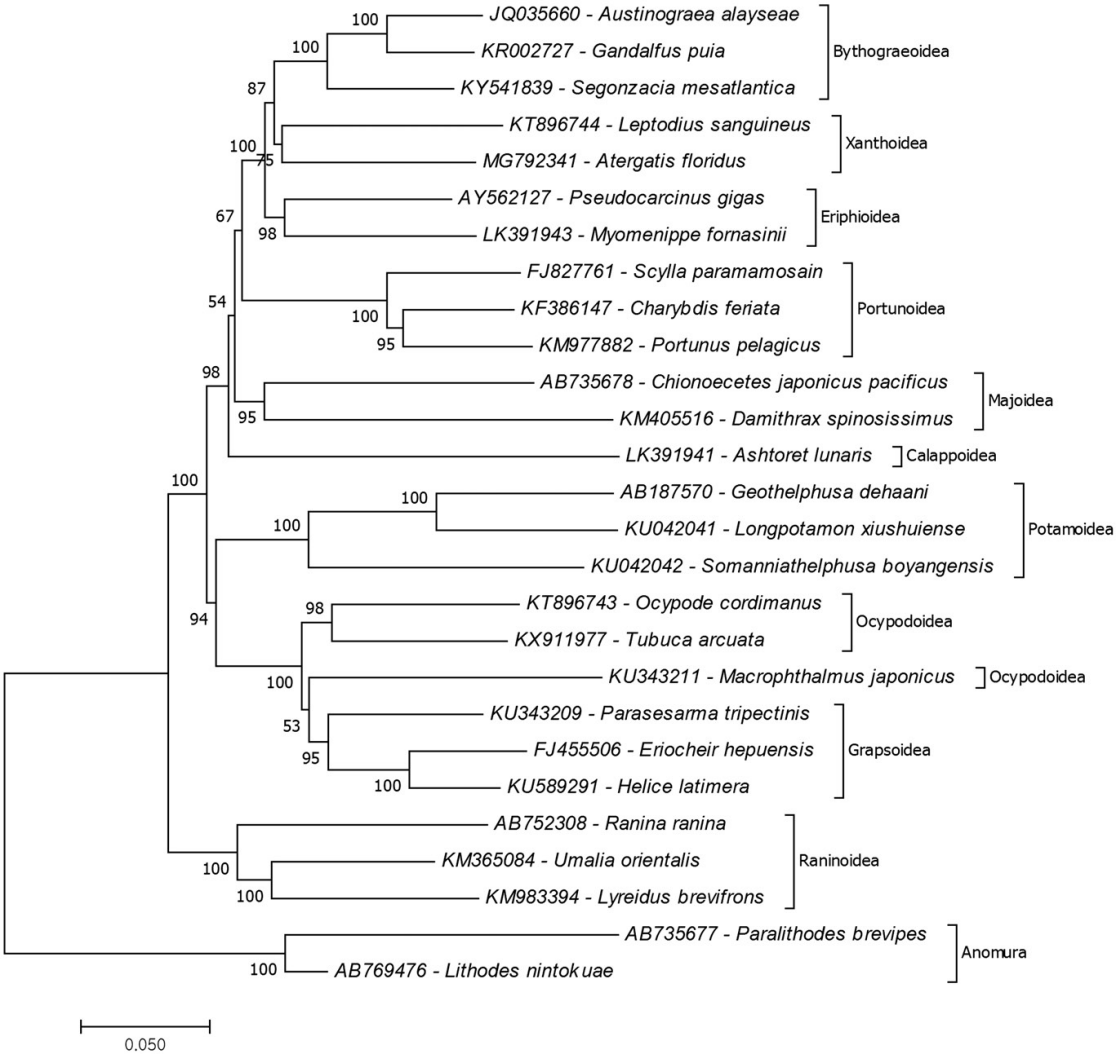
Molecular phylogeny of *A. floridus* in the infraorder Brachyura based on mitochondrial protein coding genes amino acid sequences. The data belong to *A. floridus* (MG792341) provided in the present study. The remained mitochondrial genome data retrieved form the GenBank. The species belongs to the infraorder Astacidea represents an outgroup for phylogenetic tree.
